# Saddle Pulmonary Embolus Caught in Transit across a Patent Foramen Ovale

**DOI:** 10.1155/2019/5747598

**Published:** 2019-05-02

**Authors:** Aram Barbaryan, Stefania Bailuc, Travis Abicht, Sergey Barsamyan, Yonatan Gizaw, Aibek E. Mirrakhimov

**Affiliations:** ^1^Division of General, Geriatric & Hospital Medicine, University of Kansas Health System, Kansas City, KS, USA; ^2^Department of Cardiothoracic Surgery, University of Kansas Health System, Kansas City, KS, USA; ^3^Department of Cardiology, John Radcliffe Hospital, Oxford University Hospitals NHS Trust, Oxford, UK; ^4^Division of Pulmonary and Critical Care Medicine, University of New Mexico, Albuquerque, USA

## Abstract

Impending paradoxical embolism (IPE) also described in the literature as thrombus straddling a patent foramen ovale (PFO) or paradoxical embolus in transit is a rare condition when thrombus (originating mostly in deep veins of lower extremities) embolized to the heart gets caught in PFO or in atrial septal defect without systemic embolization. We present a case of a 39-year-old female on oral contraceptive pills who presented to the emergency department with chief complaint of dyspnea and chest pain. She was found to have saddle pulmonary embolus (PE) extending through PFO to left atrium and into the left ventricle. Patient underwent emergent open pulmonary embolectomy, removal of right and left atrial thrombi, and closure of patent foramen ovale. She tolerated the surgery well and was discharged home on chronic anticoagulation therapy.

## 1. Introduction

Impending paradoxical embolism (IPE) also described in the literature as thrombus straddling a patent foramen ovale (PFO) or paradoxical embolus in transit is a rare condition when thrombus (originating mostly in deep veins of lower extremities) embolized to the heart gets caught in PFO or in atrial septal defect without systemic embolization. In 1877, Cohnheim documented the first cadaveric diagnosis of an IPE [1]. More than century later in 1985 Nellessen first described IPE using echocardiography [2]. The prevalence of PFO in adult population is 27% [3]. Normally because of left to right pressure gradient there is shunt between left and right atrium. However, in the setting pulmonary artery pressure elevation often occurs with massive and large PE that gradient reverses and the clot can migrate through PFO to left atrium causing paradoxical systemic embolization, or a clot that is larger than PFO can be trapped in PFO causing IPE [4]. We present a case of a 39-year-old female who was found to have IPE. She subsequently underwent surgical thrombectomy with excellent outcome.

## 2. Case Presentation

A 39-year-old female with history of asthma, obesity, and menorrhagia presented to the hospital with one-week history of exertional shortness of breath. In addition to dyspnea, she reported right lower extremity swelling and pain, which started 4 days prior to this presentation. The patient denied chest pain, palpitations, recent weight loss, known history of personal or family history of blood clots, history of miscarriages, and recent travel. Her only medication was oral contraceptive pill for menorrhagia.

**Figure 1:**
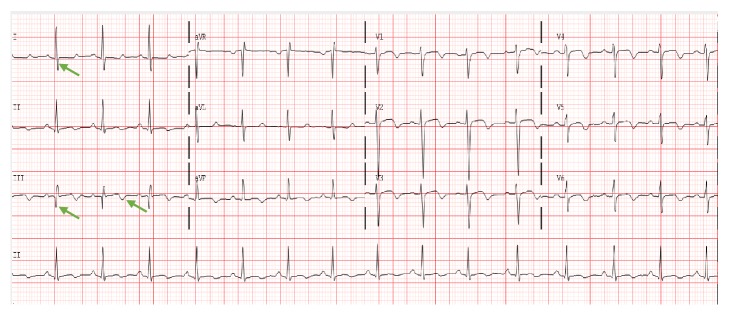
12 lead ECG on admission showing sinus rhythm 95 bpm with S_1_Q_3_T_3_ pattern (prominent S wave in lead I, Q wave and inverted T wave in lead III, green arrows).

**Figure 2:**
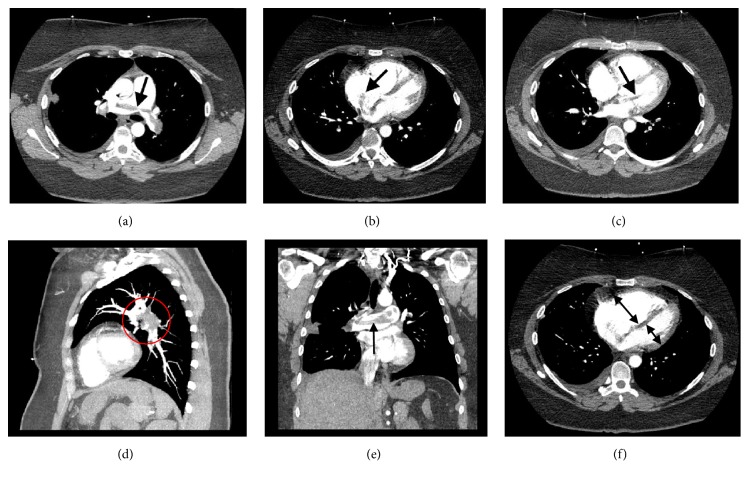
CTPA showing saddle PE (a), intracardiac thrombus extending from RA to LA through PFO (b) and further prolapsing into the left ventricle through mitral valve leaflets (c): all axial reconstruction. The same clot in sagittal view ((d), inside the red circle) and coronal view (e). RV enlargement relative to LV compressing the LV in characteristic “D shaped” pattern ((f), double arrows) all consistent with RV strain.

She was hypoxic requiring 2 liters of supplemental oxygen via nasal cannula to maintain oxygen saturation >90%, with respiratory rate 18 breaths per minute. She was mildly tachycardic with a heart rate of 108 bpm and blood pressure was 144/90, and the temperature was 36.6°C. The physical examination was significant for morbid obesity and right lower extremity swelling without sings of phlegmasia cerulea dolens. Troponin I level was normal, BNP was elevated at 704 pg/ml (reference 0-100 pg/ml), 12 lead ECG showed sinus rhythm 95 bpm with S_1_Q_3_T_3_ pattern (Figure 1): Chest X-ray was unremarkable. Wells' score was 7.5 (3 points for clinical signs and symptoms of deep vein thrombosis, 3 points of PE being the most likely diagnosis, and 1.5 points for heart rate > 100 bpm) rendering high chance of PE; therefore, CT pulmonary angiogram (CTPA) was performed (Figure 2). It demonstrated saddle type pulmonary embolus with extension into multiple segmental and subsegmental branches in the bilateral upper and bilateral lower lobes along with right ventricular (RV) strain (RV end-diastolic diameter was increased with RV to left ventricular (LV) diameter ratio >1) and mild dilatation of main pulmonary artery (35mm, normal is < 29 mm) [5–7]. CTPA also showed a linear band of low density extending within the left atrium and into the left ventricle most compatible with thrombus.

**Figure 3:**
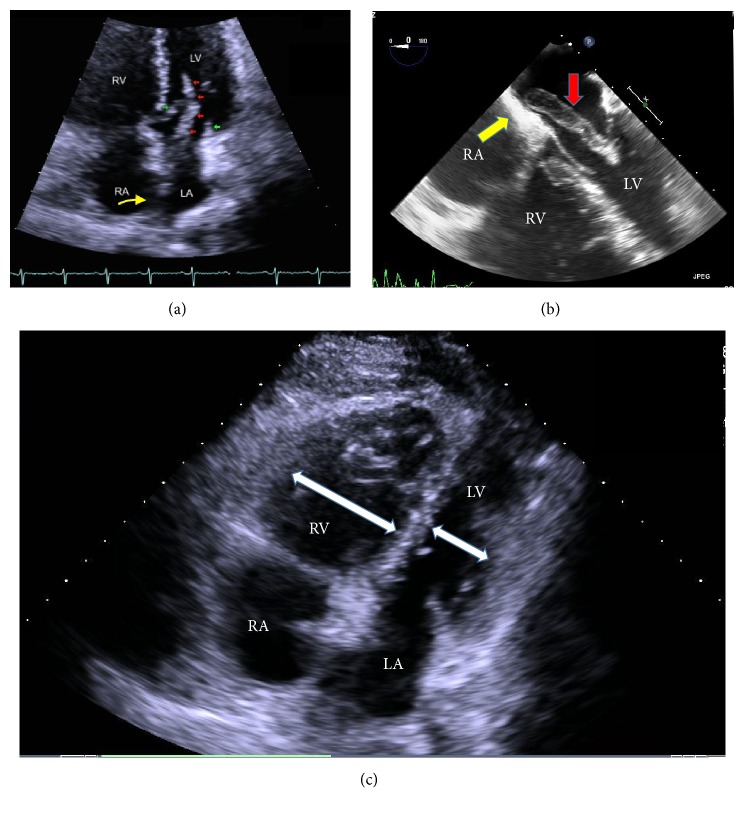
Echocardiography: (a) TTE apical four-chamber view demonstrating elongated LA thrombus (red arrows) prolapsing through PFO and LA into the LV during the diastole. Green arrows depict mitral valve leaflets. (b) The same clot is clearly visualized on TEE attached to interatrial septum. Yellow arrow depicts the location of PFO. (c) TTE subcostal view enlarged right heart chambers exceeding their left counterparts in size.

The patient was started on unfractionated heparin infusion. Transthoracic echocardiogram (TTE) was performed to further assess the intracardiac clot burden, the RV strain and to identify right-to-left shunt. TTE (Figure 3) showed a large mobile mass straddling the interatrial septum with elements in both atria, extending to and past the mitral valve and prolapsing into the left ventricle during diastole. This was highly suggestive of a communication between the atria consistent with large patent foramen ovale or atrial septal defect. Moderately dilated RV (RV/LV diameter ratio >1) with severely impaired RV systolic function, moderate RA dilation, moderate to severe tricuspid regurgitation, pulmonary artery systolic pressure of 73 mmHg and systolic flattening of the interventricular septum were also noted. All these findings were consistent with RV strain caused by PE. Lower extremity venous doppler ultrasound showed unstable occlusive extensive thrombus within the right femoral vein extending through the right popliteal vein.

**Figure 4:**
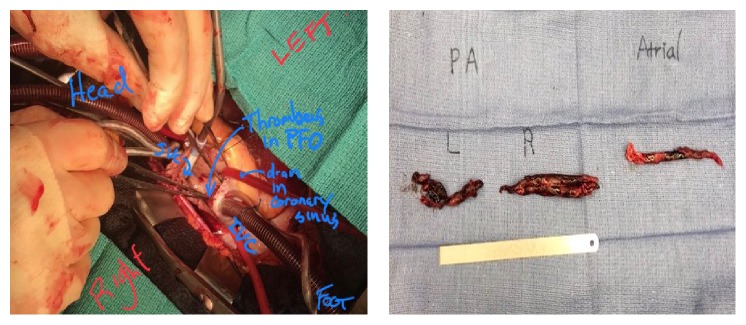
Photographs from surgical thrombectomy. Saddle pulmonary embolus was extracted from the pulmonary artery. The embolus that extended to all visualized segments of the branch pulmonary arteries was removed as well. The thrombus that was straddling the PFO was also removed en bloc. The PFO was surgically closed.

Because of large clot burden as well as impending paradoxical embolization, the patient underwent emergent open pulmonary embolectomy, removal of right and left atrial thrombi, and PFO closure (Figure 4). Intraoperative transesophageal echocardiogram (TEE) confirmed the TTE findings (Figure 3): because of the presence of an unstable extensive lower extremity venous thrombus an inferior vena cava filter was also placed. The patient tolerated the surgery well and was discharged from the hospital on oral anticoagulation with warfarin. Six month later during her follow-up visit repeat TTE demonstrated improvement in RV dilation, TV regurgitation, and PA pressure (PA systolic pressure was reduced to 26 mmHg).

## 3. Discussion

According to the International Cooperative Pulmonary Embolism Registry of 1,135 patients with PE, 42 (4%) were found to have right heart thrombus [8]. According to European Society of Cardiology 2014 guidelines on the diagnosis and management of acute pulmonary embolism, this patient is classified as having intermediate high-risk PE because of presence of RV dysfunction signs on imaging and elevated cardiac biomarkers. Intermediate risk PE patients have 30-day mortality of 3.2 - 24.5% [9–11]. Meanwhile, the presence of right heart thrombus in the setting of PE is associated with increased mortality (27-45%) with majority of fatal events occurring in the first 24 hours [8, 12]. The presence of PFO in patient with PE is an independent predictor of death, systemic embolization, and complicated hospital course [13].

Because of the complexity and heterogeneity of this patient population, the management options are individualized and no guidelines exist underlying the most cost-effective diagnostic and treatment strategies due to rarity.

Treatment can be surgical (thromboembolectomy), catheter-based (without or without thrombolysis), or conservative (systemic anticoagulation or thrombolysis). In their systematic literature review (1985-2008) that included 174 patients with IPE, Meyers et al. showed 18% mortality with cardiogenic shock and stroke among the most common causes of death. Surgical thromboembolectomy demonstrated significant decrease in composite of mortality and systemic embolization compared with anticoagulation or thrombolysis [14]. According to another systematic review surgery was associated with the lowest risk of embolization while thrombolytic therapy had the highest mortality compared with thromboembolectomy or heparin [15]. The above-mentioned findings were confirmed in the most recent literature review by Seo WW et al. that included 194 patients with thrombus trapped in PFO. In this review, surgical thrombectomy was associated with statistically significant decrease in mortality and systemic embolization compared with anticoagulation and thrombolysis. Interestingly, systemic thrombolysis was associated with higher mortality and posttreatment embolization compared with surgery and anticoagulation especially in patients without hemodynamic compromise and cardiac arrest [16].

**Figure 5:**
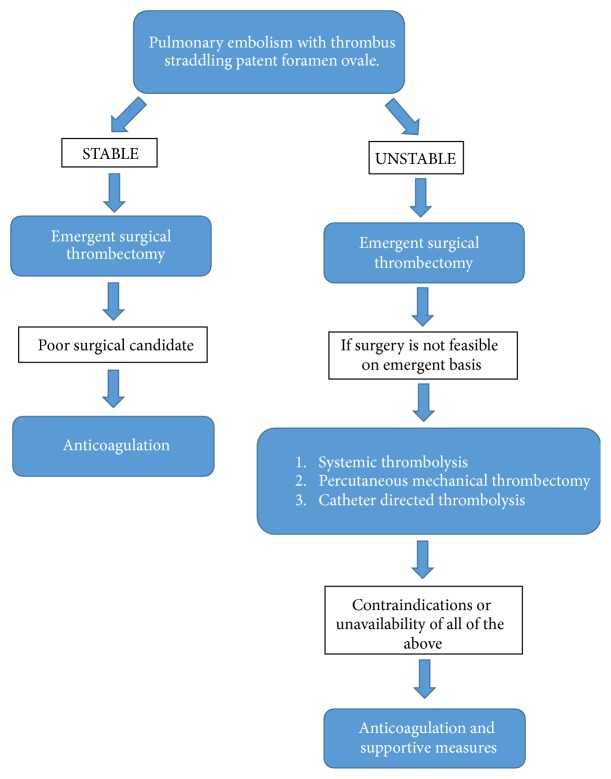


In large PE without cardiac arrest catheter directed thrombolysis (CDT) has been shown to provide similar clinical results as systemic thrombolysis but with less hemorrhagic risks because of much lower dose of thrombolytic agents (1/3 of the dose used in systemic thrombolysis) [17, 18]. However, in a patient with thrombus straddling the PFO performing CDT might disrupt the clot in RA [19]. Ultrasound-accelerated CDT (UACDT) that delivers uniform radial US energy on top of continuous small doses of thrombolytic is another approach without major bleeding complications, but it is not without risks of clot fragmentation in this patient population [20]. Percutaneous mechanical thrombectomy via clot aspiration, fragmentation, or rheolysis through high pressure saline has a success rate of up to 80% with low bleeding complications. However, in the process of clot aspiration the fragmented pieces of clot might cause systemic embolization through PFO [9]. The discussed treatment options are summarized in Figure 5 [21].

The specific treatment strategy should take into consideration individual parameters such as patient's age, hemodynamic stability, comorbidities, and bleeding risk. In general, surgical thromboembolectomy with PFO closure is favored over anticoagulation and thrombolysis, and thrombolysis is favored in hemodynamically unstable patients with contraindications to surgery [14, 15]. 2011 American Heart Association guidelines recommend echocardiographic screening for PFO in patients with massive or submassive PE (class IIb, level of evidence C). They also give class IIb, level of evidence C recommendation in favor of surgical thromboembolectomy in IPE [22].

## 4. Conclusions

Since IPE has high mortality, echocardiographic screening for PFO should be recommended in patient with massive or submassive PE. Common diseases can have rare deadly presentations. Diagnosis is easy and can significantly change the treatment strategy and improve mortality. Coordination of care and multidisciplinary team approach is important for the favorable outcome in these cases. Some medical centers employ a so-called Pulmonary Embolism Response Team (PERT), consisting of experts in cardiology, vascular surgery, pulmonary and critical care medicine, cardiac surgery, hematology, and emergency medicine [23]. Priority should be given to surgical thromboembolectomy since it has less systemic embolization compared with thrombolytic therapy and anticoagulation and it has been proven to decrease mortality.

## Abbreviations

APRN: Advanced practice registered nurse
IPE: Impending paradoxical embolism
LV: Left ventricle, left ventricular
PE: Pulmonary embolus, pulmonary embolism
PERT: Pulmonary Embolism Response Team
PFO: Patent foramen ovale
RV: Right ventricle, right ventricular
TEE: Transesophageal echocardiogram
TTE: Transthoracic echocardiogram.

## References

[1] Cohnheim I., Thrombose J., Embolie (1877). *Vorlesungen iiber Allgemeine Pathologic*.

[2] Nellessen U., Daniel W. G., Matheis G., Oelert H., Depping K., Lichtlen P. R. (1985). Impending paradoxical embolism from atrial thrombus: correct diagnosis by transesophageal echocardiography and prevention by surgery. *Journal of the American College of Cardiology*.

[3] Hagen P. T., Scholz D. G., Edwards W. D. (1984). Incidence and size of patent foramen ovale during the first 10 decades of life: an autopsy study of 965 normal hearts. *Mayo Clinic Proceedings*.

[4] Chan F. P., Jones T. R. (2001). Images in clinical medicine. Paradoxical embolus.. *The New England Journal of Medicine*.

[5] Truong Q. A., Massaro J. M., Rogers I. S. (2012). Reference values for normal pulmonary artery dimensions by noncontrast cardiac computed tomography the framingham heart study. *Circulation: Cardiovascular Imaging*.

[6] Kumamaru K. K., Hunsaker A. R., Wake N. (2012). The variability in prognostic values of right ventricular-to-left ventricular diameter ratios derived from different measurement methods on computed tomography pulmonary angiography: A patient outcome study. *Journal of Thoracic Imaging*.

[7] Kang D. K., Thilo C., Schoepf U. J. (2011). CT signs of right ventricular dysfunction: Prognostic role in acute pulmonary embolism. *JACC: Cardiovascular Imaging*.

[8] Torbicki A., Galié N., Covezzoli A., Rossi E., De Rosa M., Goldhaber S. Z. (2003). Right heart thrombi in pulmonary embolism: results from the international cooperative pulmonary embolism registry. *Journal of the American College of Cardiology*.

[9] Konstantinides S. V., Torbicki A., Agnelli G. (2014). Task Force for the Diagnosis and Management of Acute Pulmonary Embolism of the European Society of Cardiology (ESC).. 2014 ESC guidelines on the diagnosis and management of acute pulmonary embolism. *European Heart Journal*.

[10] Aujesky D., Obrosky D. S., Stone R. A. (2005). Derivation and validation of a prognostic model for pulmonary embolism. *American Journal of Respiratory and Critical Care Medicine*.

[11] Jiménez D., Aujesky D., Moores L. (2010). Simplification of the pulmonary embolism severity index for prognostication in patients with acute symptomatic pulmonary embolism. *JAMA Internal Medicine*.

[12] Rose P. S., Punjabi N. M., Pearse D. B. (2002). Treatment of right heart thromboemboli. *CHEST*.

[13] Konstantinides S., Geibel A., Kasper W., Olschewski M., Blümel L., Just H. (1998). Patent foramen ovale is an important predictor of adverse outcome in patients with major pulmonary embolism. *Circulation*.

[14] Myers P. O., Bounameaux H., Panos A., Lerch R., Kalangos A. (2010). Impending paradoxical embolism: systematic review of prognostic factors and treatment. *CHEST*.

[15] Fauveaua E., Cohen A., Bonnet N., Gacem K., Lardoux H. (2008). Surgical or medical treatment for thrombus straddling the patent foramen ovale: Impending paradoxical embolism? Report of four clinical cases and literature review. *Archives of Cardiovascular Diseases*.

[16] Seo W.-W., Kim S. E., Park M.-S. (2017). Systematic review of treatment for trapped thrombus in patent foramen ovale. *Korean Circulation Journal*.

[17] Kuo W. T., Gould M. K., Louie J. D., Rosenberg J. K., Sze D. Y., Hofmann L. V. (2009). Catheter-directed therapy for the treatment of massive pulmonary embolism: systematic review and meta-analysis of modern techniques. *Journal of Vascular and Interventional Radiology*.

[18] Kuo W. T., Banerjee A., Kim P. S. (2015). Pulmonary embolism response to fragmentation, embolectomy, and catheter thrombolysis (PERFECT): Initial results from a prospective multicenter registry. *CHEST*.

[19] Kabrhel C., Rempell J. S., Avery L. L., Dudzinski D. M., Weinberg I. (2014). Case 29-2014: A 60-year-old woman with syncope. *The New England Journal of Medicine*.

[20] Dumantepe M., Uyar I., Teymen B., Ugur O., Enc Y. (2014). Improvements in pulmonary artery pressure and right ventricular function after ultrasound-accelerated catheter-directed thrombolysis for the treatment of pulmonary embolism. *Journal of Cardiac Surgery*.

[21] Nakamura K., Alba G. A., Scheske J. A. (2016). A 57-year-old man with insidious dyspnea and nonpleuritic chest and back pain. *CHEST*.

[22] Jaff M. R., McMurtry M. S., Archer S. L. (2011). Management of massive and submassive pulmonary embolism, iliofemoral deep vein thrombosis, and chronic thromboembolic pulmonary hypertension: a scientific statement from the american heart association. *Circulation*.

[23] Dudzinski D. M., Piazza G. (2016). Multidisciplinary pulmonary embolism response teams. *Circulation*.

